# Pulmonary Function and Incidence of Selected Respiratory Diseases Depending on the Exposure to Ambient PM_10_

**DOI:** 10.3390/ijms17111954

**Published:** 2016-11-22

**Authors:** Artur Badyda, Anna Gayer, Piotr Oskar Czechowski, Grzegorz Majewski, Piotr Dąbrowiecki

**Affiliations:** 1 Faculty of Building Services, Hydro- and Environmental Engineering, Warsaw University of Technology, 20 Nowowiejska St., PL00-653 Warsaw, Poland; artur.badyda@is.pw.edu.pl; 2 Department of Information Systems, Gdynia Maritime University, 83 Morska St., PL81-225 Gdynia, Poland; oskar@am.gdynia.pl; 3 Faculty of Civil and Environmental Engineering, Warsaw University of Life Sciences, 159 Nowoursynowska St., PL02-776 Warsaw, Poland; grzegorz_majewski@sggw.pl; 4 Military Institute of Medicine, Central Clinical Hospital of the Ministry of National Defense, 128 Szaserów St., PL04-141 Warsaw, Poland; pdabrowiecki@wim.mil.pl; 5 Polish Federation of Asthma, Allergy and COPD Patients’ Associations, 23/102 Łabiszyńska St., PL03-204 Warsaw, Poland

**Keywords:** pulmonary function, respiratory diseases, ambient air pollution, PM_10_

## Abstract

It is essential in pulmonary disease research to take into account traffic-related air pollutant exposure among urban inhabitants. In our study, 4985 people were examined for spirometric parameters in the presented research which was conducted in the years 2008–2012. The research group was divided into urban and rural residents. Traffic density, traffic structure and velocity, as well as concentrations of selected air pollutants (CO, NO_2_ and PM_10_) were measured at selected areas. Among people who live in the city, lower percentages of predicted values of spirometric parameters were noticed in comparison to residents of rural areas. Taking into account that the difference in the five-year mean concentration of PM_10_ in the considered city and rural areas was over 17 μg/m^3^, each increase of PM_10_ by 10 μg/m^3^ is associated with the decline in FEV_1_ (forced expiratory volume during the first second of expiration) by 1.68%. These findings demonstrate that traffic-related air pollutants may have a significant influence on the decline of pulmonary function and the growing rate of respiratory diseases.

## 1. Introduction

Exposure to ambient air pollution, especially to particulate matter, is a major risk factor for pulmonary diseases such as asthma, chronic bronchitis or pneumonia [1,2]. Numerous epidemiological studies indicate that long- and short-term exposure to PM_10_ (particulate matter with an aerodynamic diameter equal to or less than 10 µm) cause increased morbidity and mortality [3,4,5,6,7]. PM_10_ penetrates deeply into the respiratory system, causing acute and chronic effects by increasing oxidative stress, inflammation, and cytotoxicity [8,9].

The number of hospital admissions for cardiovascular and respiratory disorders was used as an indicator of the particulate matter influence on human health. Eight European cities—the APHEA 2 project (Air Pollution on Health: European Approach) in which short-term health effects of particulate matter have been investigated—confirmed that particle concentrations in these cities were positively associated with increased numbers of admissions for pulmonary diseases [10]. The burden of chronic diseases due to air pollution is still not as well defined as the acute effects. Exposure to air pollution may cause exacerbations of pre-existing asthma as well as contribute to the onset of new cases. The results of a study on 10 European cities attributed exposure to traffic-related air pollutants with 15% of childhood asthma exacerbations and 14% of the cases of incidence of asthma [11].

A study conducted by Zanobetti et al. [12] in 10 US cities indicated that a daily 10 μg/m^3^ increase of PM_10_ resulted in a 2.5% (95% confidence interval (CI): 1.8–3.3) increase in chronic obstructive pulmonary disease, a 1.95% (95% CI: 1.5–2.4) increase in pneumonia, and a 1.27% increase (95% CI: 1–1.5) in cardiovascular diseases.

Cross-sectional studies by Schikowski et al. [13] showed a decline in FEV_1_ (forced expiratory volume during the first second of expiration), and the development of chronic obstructive pulmonary disease (COPD) as associated with urban traffic-related PM_10_. Using GOLD (Global Initiative for Chronic Obstructive Lung Disease) criteria (FEV_1_/FVC ratio < 0.7 is the main criterion for COPD), they demonstrated that an increase of 7 μg/m^3^ ambient PM_10_ (over five years) was associated with a 5.1% decline in FEV_1_ and with a 1.33 increased risk of COPD.

It is essential in pulmonary disease research to take into account traffic-related air pollutant exposure among urban inhabitants. According to epidemiological studies conducted in several European cities, approximately 31% of the population live within 75 m of busy roads, and 53% within 150 m [14]. That makes traffic-related pollution exposure and changes in its concentration in ambient air substantial when concerning its impact on human health. Not only can increasing air pollutant concentrations be associated with negative health effects, but also a decrease in ambient PM_10_, as Schindler et al. [15] found, has positive health outcomes. A Swiss adult cohort study shows that a decrease in the concentration of particulate matter was associated with a reduction of respiratory symptoms. Another cohort study [16] concerning children’s exposure to urban air pollution demonstrated that moving to a less polluted area both can accelerate their lung function growth and, if previous impairments occurred, they could be reversed.

Long-term exposure to PM_10_ associated with incident cases of lung cancer was observed among over 103,000 women from the Nurses’ Health Study (NHS) cohort [17]. During the study time, 2155 of them were diagnosed with lung cancer. Those findings indicate an increased risk of incident lung cancer associated with ambient PM exposure both among never and long-term former smokers. A 10 μg/m^3^ increase in a 72-month-average PM_10_ (hazard ratio (HR) = 1.04; 95% CI: 0.95, 1.14) was positively associated with lung cancer.

## 2. Results

Traffic-related air pollutant concentrations were statistically significantly higher (*p* < 0.001) in the vicinity of roads in comparison to the rural areas, which was confirmed by all of the tests (Mann–Whitney *u*-test, Wald–Wolfowitz test and Kolmogorov–Smirnov test). Table 1 presents the mean values of concentrations of selected air pollutants in Warsaw (average concentration from seven locations) and control areas (average concentration from two locations). It should also be mentioned that the five-year mean concentrations of PM_10_ in the same areas were statistically significantly different (*p* < 0.001) as well and were equal to 39.8 μg/m^3^ (±7.2) in urban area (Warsaw) and 22.1 μg/m^3^ (±1.5) in the control areas (medians amounted to 37.2 and 22.8 respectively).

In the short-term measurements, only in Warsaw were exceedances of daily PM_10_ limit values found. In the long-term air quality monitoring results, received from the National Environmental Monitoring system, the PM_10_ concentrations above the daily limit values concerned both Warsaw and the control area. However, the average number of times when the daily limit value was exceeded in a calendar year varied in Warsaw from 63 to 97 (on average, depending on the year), but in the control areas it varied from 9 to 15, which is within the acceptable range of up to 35 exceedances in a calendar year. Also, the mean annual concentration of PM_10_ in Warsaw was significantly higher than in the control areas—the average yearly concentrations in Warsaw in three of five years revealed an exceedance of the limit value, and in the remaining two years were only slightly lower than the limit value. In the control locations, the mean annual concentration accounted for little more than half of the limit value. Details are presented in Table 2.

The significant association between living close to a busy road and the relative risk (RR) of obstruction was found and was particularly high among non-smoking persons. The RR of obstruction occurrence in the city group in comparison to the control group was at the level of 4.1. Statistically significant (*p* < 0.001) differences in the distributions of FEV_1_, PEF (peak expiratory flow—the maximum velocity of flow measured during forced exhalation) and MEF_50_ (maximal expiratory flow at 50% of vital flow capacity) were observed between inhabitants of Warsaw and the control group. However, in general, the mean values of the percentages of predicted values of spirometric parameters met the normative values [18].

Hereafter, the focus was primarily on FEV_1_ changes and particulate matter pollution. As was already shown by Badyda et al. [19], the decrease in the absolute mean value of FEV_1_ in the city group compared to the control group was at the level of 2.97%. When, however, the percentages of predicted values were taken into account, the differences in the decline reached 3.65%. In the group of non-smokers, the differences were higher and amounted to 3.09% in the case of absolute values and 5.00% in the percentages of predicted values. Similar differences were observed also in the case of other spirometric parameters that were considered in the investigation, except the FVC (forced vital capacity—the capacity of air which is exhaled by a tested person during a forced exhalation after maximum slow inhalation), with respect to which the differences were also observed but they were statistically insignificant (*p* > 0.05). Details of these results are presented in Table 3 and Table 4.

**Figure 1:**
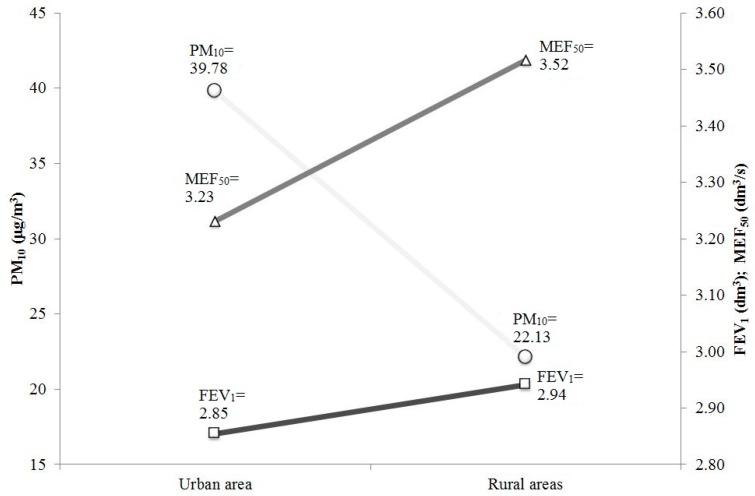
Mean values of FEV_1_ (forced expiratory volume during the first second of expiration) and MEF_50_ (maximal expiratory flow at 50% of vital flow capacity) in the city and control group and five-year average concentration of PM_10_ in the air of urban and rural areas.

Taking into account that the difference in the five-year mean concentration of PM_10_ in the considered city and rural areas was over 17 μg/m^3^, each increase of PM_10_ by 10 μg/m^3^ is associated with a decline in FEV_1_ of 1.68%, in PEF of 1.18% and in MEF_50_ of 4.61%, considering that the last two parameters have less clinical relevance in terms of the influence on air pollution (selected results are presented in Figure 1). The differences in the concentrations of PM_10_ and in the spirometric parameters between the groups were statistically significant, which confirms that traffic and its air pollutants may have a substantial influence on the pulmonary function of people living in particularly polluted areas.

Substantial differences were observed when taking into account the differences between city and control groups in men and women. The reduction of spirometric parameters was considerably higher in women, in whom the increase in PM_10_ of 10 μg/m^3^ was associated with a decline in FEV_1_ of 4.22%, in PEF of 2.15% and in MEF_50_ of 6.97%, while in men the decrease in FEV_1_ was only of 0.62% and in MEF_50_ of 4.38%. In the case of PEF, a slight increase of 1.15% was observed. The differences between men and women could be associated with sport activities, which were noticeably more frequently declared by men in the city group in comparison to the control group, whereas among women the difference in sport activity declarations was smaller.

**Figure 2:**
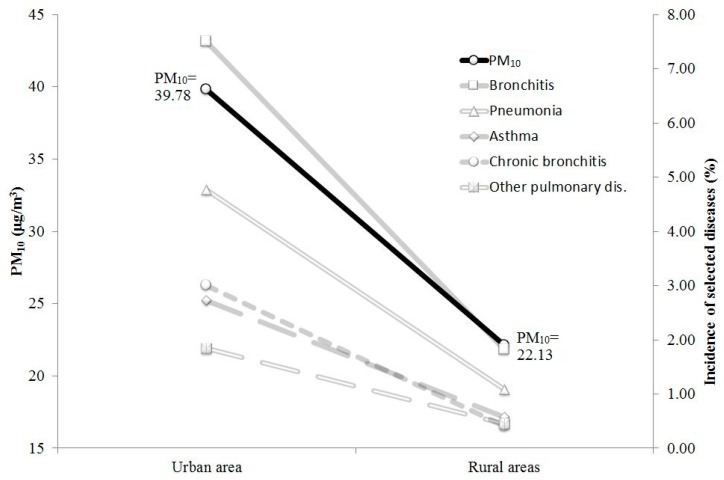
Incidence of selected respiratory diseases in the city and control groups on a background of the five-year mean concentration of PM_10_ in the urban area and considered rural areas.

It also seems that the exposure to higher concentrations of PM_10_ may have an influence on the incidence of respiratory diseases. In the city group, according to the results of the Fisher-Snedecor test, the proportion of the incidence of considered diseases was statistically significantly higher in comparison to the control group (Figure 2).

In the whole investigated group, a 10 μg/m^3^ increase of the PM_10_ concentration was associated with an increase of the incidence of the diseases. The values given below are the multiplicities of the occurrence of particular diseases associated with the rise of the five-year average concentration of PM_10_ in the ambient air by each 10 μg/m^3^:

- 4.23 in case of chronic bronchitis;
- 2.71 in case of asthma;
- 2.50 in case of pneumonia;
- 2.35 in case of other pulmonary diseases (including COPD);
- 2.33 in case of bronchitis.

Similar situations such as the case of differences in spirometric parameters between men and women were observed in the case of the incidence of diseases. The differences were higher in women. The incidence associated with an increase of PM_10_ by 10 μg/m^3^ was as follows:

- In case of chronic bronchitis: 4.37 in women and 3.85 in men;
- In case of asthma 4.64 in women and 1.57 in men;
- In case of pneumonia 2.50 in women and 2.48 in men;
- In case of other pulmonary diseases (including COPD) 4.05 in women and 1.50 in men;
- In case of bronchitis 2.65 in women and 1.81 in men.

## 3. Discussion

The study shows that a 10 μg/m^3^ increase in the PM_10_ concentration is associated with adverse health effects to the human respiratory system. Similar studies taking into account a 10 μg/m^3^ increase of PM_10_ in short- and long-term exposure and the respiratory effects were conducted worldwide. Hoek et al. [20] assembled results from cross-sectional studies from 12 countries which included the assessment of respiratory health and exposure to particulate air pollution data of over 45,000 children. They found positive associations between the average PM_10_ concentration and lung impairment symptoms such as the prevalence of phlegm (odds ratio (OR) per 10 μg/m^3^: 1.15, 95% CI: 1.02–1.30), hay fever (OR: 1.20, 95% CI: 0.99–1.46), bronchitis (OR: 1.08, 95% CI: 0.98–1.19), morning cough (OR: 1.15, 95% CI: 1.02–1.29) and nocturnal cough (OR: 1.13, 95% CI: 0.98–1.29).

The most common findings in that field come from differences in the number of hospital admissions for respiratory diseases due to changes in ambient particulate matter exposure. In a multi-city study [21] conducted in 36 US cities, a 0.79% increase in pneumonia admissions was associated with a 10 μg/m^3^ increase in PM_10_ during the warm season. A Chinese study [22] conducted in 15 major hospitals of Hong Kong showed that for every 10 μg/m^3^ increase in NO_2_, O_3_ and PM_2.5_ in subjects aged older than 65 years, the RR for asthma hospitalization was 1.02.

In APHEA, a 2% change in the mean number of daily admissions per 10 μg/m^3^ increase was as follows: asthma (0–14 years) 1.2% (95% CI: 0.2, 2.3), asthma (15–64 years) 1.1% (0.3, 1.8), and COPD plus asthma and all-respiratory (>65 years) 1.0% (0.4, 1.5) and 0.9% (0.6, 1.3) [10]. Differentiation in adverse health effects between two groups exposed to different PM concentrations, as assumed by the authors, can be calculated from the proportion of airflow abnormalities. As Götschi et al. [23] stated, the pulmonary function test is a widely used preclinical marker of chronic disease and lung function may be considered as one of the strongest predictors of life expectancy.

The Health Survey for England (HSfE) study [24] which took into account almost 10 times more spirometry results (42,975) than in this study shows similar associations between the increase in PM concentration (with a diameter <10 μm) and lower FEV_1_ (3% decrease in HSfE, 1.68% in Warsaw study).

Numerous studies confirm that the prevalence of respiratory symptoms is associated with the place of residence and the distance from roads with a high traffic volume. Results obtained by McConnell et al. [25] indicate that an increased risk of asthma (OR: 1.29, 95% CI: 1.01, 1.86) was associated with residing within a distance of 75 m from a major road. This might strengthen the results of the presented study, as the “city group” with worse respiratory parameters consists of residents who live close to major roads.

According to gender analytic studies conducted by Clougherty [26], women are more susceptible to adverse respiratory health effects due to their exposure to air pollution. This correlation is also confirmed by the presented study where differences between men and women in spirometric parameters occurred. In our study, the incidence of respiratory diseases associated with an increase in PM_10_ of 10 μg/m^3^ was higher among woman.

The main strength of the study was the wide range of considered diseases, whereas other studies are mainly focused on incidents of COPD [27] and asthma [28].

Another strength of the study is the selection of the investigated cases. In the following study, only never smokers were taken into account. Among smokers and ex-smokers, the prevalence of respiratory diseases is higher because of the negative effects of tobacco smoke. That factor prevails even when exposure to ambient air pollution is taken into account. Among never smokers, tobacco smoke as a confounding element is excluded. Several studies show that respiratory diseases can be diagnosed also among non-smokers, who are at risk of other factors including exposure to air pollutants. Behrendt et al. [29] found that a quarter of COPD cases in the US were diagnosed in never smokers. Similar findings indicate that in the UK [30] it is 22.9%, and in Spain it is 23.4% [31].

There are also some limitations with regard to the presented research. As no data from indoor exposure was available, an important limitation of the study is that it was not able to take into account indoor air pollution as a risk factor for respiratory impairments. The pulmonary function test was made once in each patient, so there is a need for a follow-up study.

Even though there are numerous studies based only on particulate matter exposure, the results obtained by Janssen et al. [32] showed that the health effects of a 1 μg/m^3^ increase in exposure were greater for black carbon particles (BCP) than for PM_10_ or PM_2.5_. They suggest that the effect of BCP is more robust than the effect of particulate matter mass. This should be considered in subsequent studies.

## 4. Materials and Methods

This research was conducted in the years 2008–2012 and 4985 people took part. They were recruited from two types of locations: inhabitants of Warsaw (Poland) living in the proximity of seven selected roads characterized with high traffic density (city group) and residents of two selected rural areas characterised by one of the lowest concentrations of air pollutants (mainly particulate matter) in the country, isolated from the direct influence of principal air pollutants emission sources (control group). In order to avoid the influence of air pollutants originating from other sources than vehicular traffic (low-stack emission sources in particular), and thus to affect the representativeness of the sample, spirometric tests were performed between April and June and between September and October.

Traffic density, traffic structure and velocity as well as concentrations of selected air pollutants (CO, NO_2_ and PM_10_) were measured in the cross sections of above-mentioned streets. We used two different ways of gathering data on air quality. The short-term measurement were completed using the compact, semi-mobile air quality monitoring station Airpointer^®^. Considered air pollutants were measured using the following measurement principles:

- nitrogen dioxide: chemoluminescence method (EN14211);
- carbon monoxide: NDIR gas filter correlation (EN14626);
- particulate matter PM_10_: nephelometry.

One-week measurement sessions (in the city within seven weeks in total and in the rural areas within four weeks in total) were completed in June and September (in order to exclude or at least decrease the influence of other sources of air pollutants emission, in particular the municipal and household sources), so in the similar periods to the execution of pulmonary function tests. One-minute concentrations were collected and aggregated automatically to 1-h average concentrations. Results presented in Table 1 are averaged data for the entire duration of the air pollutants concentrations measurement. On the basis of these data we assessed the air quality in the exact places, where the medical examinations were then conduced. Due to some limitations (including financial) long-term measurements with the use of Airpointer^®^ were not possible to be implemented.

Besides, data on concentration of PM_10_ for years 2008–2012 from Air Quality Monitoring System (three stations in Warsaw and two stations in rural areas) were also collected in order to compare the long-term exposure of city and rural areas residents. By the reason of sparse deployment of air quality monitoring stations in Poland, it was not possible to use the results of continuous measurements in the exact locations which were subject to medical investigations. In particular, this concerned the location where the control group was examined. Therefore, using the practice of the Inspection for Environmental Protection, an assumption was made, that the exposure to PM_10_ of the study subjects is as it results from the measurements conducted at monitoring stations located closest to the investigation sites. The distance between examined rural residents house and monitoring station was 0.1–30 km and for urban residents 0–5 km. The General Inspectorate for Environmental Protection has made available us the data on the average 1-h concentrations of PM_10_ from three monitoring stations located in Warsaw and two stations located in the vicinity of the control group study location. On the basis of these data, annual average concentrations were calculated, which for the needs of this paper were aggregated to five-year mean values.

In the final analyses 4725 people were included. People who did not properly cooperate with the doctor/nurse during the pulmonary function test and patients already treated for COPD or bronchial asthma, were disqualified from further analyses. Basic characteristic information concerning both groups are presented in Table 5.

By reason of the aim of the research, which was the assessment of traffic-related air pollutants impact on pulmonary function, the results of non-smoking people only were taken into account in the analyses. Details of the medical examination were described by Badyda et al. [33]. In general the examination was made up of:

- information on the aim of the pulmonary function test and lack of its adverse effects on human health;
- subjective research (questionnaire);
- objective research (pulmonary function test), which included the measurement of forced vital capacity (FVC), forced expiratory volume during the first second of expiration (FEV_1_), forced expiratory flow at 50% of FVC (FEF_50_), peak expiratory flow (PEF) and the calculation of the pseudo-Tiffeneau factor (FEV_1_/FVC).

The differences in FEV_1_ (as the major parameter determining the occurrence of bronchi obstruction) and the incidence of selected respiratory diseases between the city group and control group were calculated in relation to five-year mean concentration of PM_10_.

The final confirmation of the initially observed dependencies and mutual relationships were selected statistical models. Kolmogorov-Smirnov test with the Liliefors amendment and Shapiro-Wilk tests were used to the evaluation of the normal distribution and the Levene test to check the homogeneity of variance. All of the results were considered as statistically significant when the probability of error was *p* < 0.05. Due to the rejection of the hypothesis of normality of specific distributions the comparison was made using both classical parametric tests, as the analysis of variance (ANOVA) with one-way classification, as well as non-parametric methods, as the Kruskal–Wallis test, Mann–Whitney *u*-test and Wald–Wolfowitz test.

The significance of differences in the proportion of airflow abnormalities occurrence and pulmonary diseases incidence between two analysed groups were compared using the test of differences between the two indicators of structure. When the multiple proportion (more than two) were compared, the Fisher-Snedecor test was used [34]. The objective of the test is to investigate the assumption that the proportions *p_1pr_*, *p_2pr_* and *p_kpr_* of elements from *k* populations are equal, based on *k* independent samples, one from each population: $H_{0}:p_{1}=p_{2}=p_{3}\ldots p_{k}$ and $H_{1}:\neg H_{0}$. The limitation of the test is that it is approximate and assumes that the number of observations in the samples is sufficiently large (i.e., *n_1_*, *n_2_*, …, *n_k_* ≥ 20). Two test statistics, depend on proportions size: (1) $0.2\leq p_{pr}\leq 0.8$ and (2) $p_{pr}>0.8\;or\;p_{pr}<0.2$. These statistics are as follows:

(1). $F=\frac{\sum n_{i}{\left(p_{ipr}-\bar{p}\right)}^{2}}{\sum p_{ipr}q_{ipr}}\cdot \frac{k}{k-1}$ with Fisher distribution, where $v_{1}=k-1$; $v_{2}-\infty$

(2). $F=\sum n_{i}{\left(x_{i}-\bar{x}\right)}^{2}\cdot \frac{1}{k-1}$, where: $x_{i}=2\;arcsin\sqrt{p_{ipr}}$ and $\bar{x}=2\;arcsin\sqrt{\bar{p}}$ with Fisher distribution, where $v_{1}=k-1$; $v_{2}-\infty$

The statistical analyses were performed with the use of STATISTICA 10 software.

## 5. Conclusions

People living in urban areas, being exposed to statistically significantly higher concentrations of traffic-related air pollutants (PM_10_ in particular), are characterized by substantially lower percentages of predicted values of spirometric indices in comparison to residents of rural areas. Exposure to PM_10_ is also associated with the decrease of absolute values of spirometric parameters (including FEV_1_, MEF_50_ and PEF) and the increase of the incidence of several respiratory diseases. These findings demonstrate that traffic-related air pollutants may have a significant influence on the decline of pulmonary function and the growing rate of respiratory diseases such as bronchitis, pneumonia, bronchial asthma or COPD.

## Figures and Tables

**Table 1:** Basic statistics of concentrations of selected air pollutants measured in urban and control areas during short measurements.

Air Pollutant	Urban Area		Control Area		*p*
	Mean ± SD	Median	Mean ± SD	Median	
CO (μg/m^3^)	1308.2 ± 687.4	1262.7	451.5 ± 45.0	444.5	*p* < 0.001
NO_2_ (μg/m^3^)	52.7 ± 29.8	47.6	1.3 ± 0.7	1.3	
PM_10_ (μg/m^3^)	42.7 ± 10.7	39.1	7.2 ± 2.3	6.5	

Mean: average yearly concentration calculated on 1 h concentrations; SD: standard deviation; *p*: level of significance.

**Table 2:** Basic statistics of annual PM_10_ concentrations in years 2008-2012 in urban and control areas measured in air quality monitoring stations of the National Environmental Monitoring.

Year	Loc	Number of Data	Mean	Min	Max	SD	Mean in S	Mean in W	Number > 50
2008	UA	8428	35.9	1.0	411.4	24.1	33.4	38.1	62
	CA	8489	22.8	0.2	254.0	15.7	22.3	23.3	9
2009	UA	8465	40.3	1.7	475.4	29.1	35.1	45.8	84
	CA	8122	23.4	1.0	404.0	19.4	23.1	24.1	12
2010	UA	7816	42.9	1.9	366.1	30.5	32.3	50.3	97
	CA	7421	23.2	0.9	220.2	17.8	19.1	26.9	10
2011	UA	8392	40.6	2.5	360.7	27.8	32.5	48.9	81
	CA	6817	21.4	0.0	290.1	19.8	17.3	22.4	15
2012	UA	8040	39.3	0.8	433.6	28.2	30.4	45.5	70
	CA	8411	20.1	0.0	213.6	17.4	18.1	22.7	9

Loc: location of the measurement; UA: urban area; CA: control area; Min: minimum 1 h concentration in the calendar year; Max: maximum 1 h concentration in the calendar year; Mean in S: average concentration in the summer half of year; Mean in W: average concentration in the winter half of year; Number > 50: average number of days in calendar year with the exceedance of PM_10_ daily limit value (50 μg/m^3^).

**Table 3:** Mean values and standard deviations of the absolute values of basic spirometric parameters in the city and control group.

Variable	City Group	Control Group	*p*
FEV_1_ (dm^3^)	2.85 ± 1.10	2.94 ± 0.99	*p* < 0.05
PEF (dm^3^/s)	7.11 ± 2.54	7.27 ± 2.40	
MEF_50_ (dm^3^/s)	3.23 ± 1.73	3.52 ± 1.67	

FEV_1_: forced expiratory volume during the first second of expiration; PEF: peak expiratory flow; MEF_50_: maximal expiratory flow at 50% of vital flow capacity.

**Table 4:** Mean values and standard deviations of the percentages of predicted values of basic spirometric parameters in the city and control group.

Variable	City Group	Control Group	*p*
FEV_1_ (%)	94.26 ± 19.30	97.82 ± 18.30	*p* < 0.001
PEF (%)	95.95 ± 23.93	99.14 ± 22.70	*p* < 0.01
MEF_50_ (%)	73.97 ± 33.04	82.42 ± 33.10	*p* < 0.001

**Table 5:** Elementary information on city and control groups.

Variable	City Group	Control Group
Number of patients	3834	891
Men/Women	1608/2226	420/471
Smokers/Non-smokers	1896/1938	442/449
